# Human Cumulus Cells Molecular Signature in Relation to Oocyte Nuclear Maturity Stage

**DOI:** 10.1371/journal.pone.0027179

**Published:** 2011-11-07

**Authors:** Zamalou Gisèle Ouandaogo, Delphine Haouzi, Said Assou, Hervé Dechaud, Issac Jacques Kadoch, John De Vos, Samir Hamamah

**Affiliations:** 1 CHU Montpellier, Institute for Research in Biotherapy, Hôpital Saint-Eloi, INSERM U1040, Montpellier, France; 2 Université MONTPELLIER1, UFR de Médecine, Montpellier, France; 3 ART/PGD Department, CHU Montpellier, Hôpital Arnaud de Villeneuve, Montpellier, France; 4 Département d'Obstétrique Gynécologie, Université de Montréal, Hopital Saint-Luc du CHUM, Montréal, Canada; University of California San Diego, United States of America

## Abstract

The bi-directional communication between the oocyte and the surrounding cumulus cells (CCs) is crucial for the acquisition of oocyte competence. We investigated the transcriptomic profile of human CCs isolated from mature and immature oocytes under stimulated cycle. We used human Genome U133 Plus 2.0 microarrays to perform an extensive analysis of the genes expressed in human CCs obtained from patients undergoing intra-cytoplasmic sperm injection. CC samples were isolated from oocyte at germinal vesicle, stage metaphase I and stage metaphase II. For microarray analysis, we used eight chips for each CC category. Significance analysis of microarray multiclass was used to analyze the microarray data. Validation was performed by RT-qPCR using an independent cohort of CC samples. We identified differentially over-expressed genes between the three CC categories. This study revealed a specific signature of gene expression in CCs issued from MII oocyte compared with germinal vesicle and metaphase I. The CC gene expression profile, which is specific of MII mature oocyte, can be useful as predictors of oocyte quality.

## Introduction

The bidirectional exchanges between oocyte and contiguous CCs are important for oocyte competence acquisition, early embryonic development and CC expansion [1]–[3]. Oocyte maturation starts with the resumption of the first meiosis process, and is divided in nuclear and cytoplasmic maturation. During oocyte nuclear maturation, there is progression from prophase I characterized by germinal vesicle breakdown (GVBD) to metaphase II (MII) of the second meiosis [2], [4]. At the end of this process, the oocyte should be considered as mature and able to be fertilized. However, the main problem, which hinders IVF/ICSI success, is how to select oocytes competent for embryonic development and implantation. Gene expression profile of CCs has been suggested to predict embryo development and pregnancy outcome [5]–[12]. However, in the majority of these studies, they did not consider the possibility that CC gene expression profile might vary according to the stages of oocyte nuclear maturation and thus were focused mostly on a single specific phase of oocyte maturation, such as the MII stage [6]. In humans, it is not known whether MII oocytes are systematically surrounded by specific CC molecular signature. Hence, the objective of the present study was to investigate gene expression profiles of human CCs isolated from oocytes at the germinal vesicle (CC_GV_), metaphase I (CC_MI_) and metaphase II (CC_MII_) stage, under controlled ovarian stimulation (COS) cycle and to evaluate the % of MII mature oocyte surrounded by mature CCs. This study has been performed by microarray analysis in order to identify potential biomarkers related to oocyte nuclear maturity and/or oocyte quality.

## Materials and Methods

### Processing of cumulus cells

Normal responder patients (age<36) referred to our center for intra-cytoplasmic sperm injection (ICSI) were included in this study after written informed consent. This project was approved by the Institute Review Board. Patients were stimulated with a combination of GnRH agonist or antagonist protocols with recombinant FSH or with HP-hMG. COCs were recovered under ultrasound echo-guidance 36 h after human Chorionic Gonadotrophin (5 000 UI, hCG) administration. CCs were separated mechanically from the corresponding oocyte as previously described [8]. A total of 111 CC samples obtained from 40 patients were used in this study.

For microarray analyses, 24 individual CC samples obtained from 16 patients were issued from COC (i) at germinal vesicle stage, (ii) metaphase I stage, and (iii) metaphase II stage. The differential gene expression profile in the three CC groups was investigated. For reverse-transcription quantitative polymerase chain reaction (RT-qPCR), 24 CC samples (8 samples for each stage of nuclear maturation) obtained from 19 patients were used.

For evaluating the reliability of the specific MII CC molecular signature, we tested this molecular signature on 53 CC samples isolated from mature (MII) oocytes issues from patients underwent ICSI procedure for male infertility (n = 5).

### Complementary RNA preparation and microarray hybridization

Total RNA from CC samples was extracted using the RNeasy Micro Kit (Qiagen). RNA was quantified using a Nanodrop ND-1000 spectrophotometer (Nanodrop Technologies, Wilmington, DE, USA). RNA integrity and quality were evaluated with an Agilent 2100 Bioanalyzer (Agilent, Palo Alto, CA, USA). RNA samples were stored at −80°C until microarray analysis. The Affymetrix 3′ IVT express protocol (ref 901229) was used to prepare cRNA (one-cycle amplification) with a starting concentration of 100 ng of total RNA. First-strand DNA was synthesized using an oligo-dT primer that incorporates a T7 promoter sequence. cDNA was then amplified by in vitro transcription (IVT) with T7 RNA polymerase. During RNA amplification (aRNA) a biotinylated nucleotide analog was incorporated to be used as a label for the message. After fragmentation, the labeled anti-sense aRNA was hybridized to HG-U133 Plus 2.0 arrays (Affymetrix™) as described previously [13].

### Data processing

Scanned GeneChip images were processed using the Affymetrix GCOS 1.4 software. Microarray data were analyzed using the Affymetrix Expression Console™ software and normalization was performed with the MAS5.0 algorithm to obtain the signal intensity and the detection call (present, marginal, or absent) for each probe set. This algorithm determines whether a gene is expressed with a defined confidence level or not (“detection call”). This “call” can either be “present” (when the perfect match probes are significantly more hybridized than the mismatch probes, FDR<0.04), “marginal” (for FDR≥0.04 and ≤0.06) or “absent” (FDR>0.06). FDR, false discovery rate. The data are accessible at the Gene Expression Omnibus (GEO) through the provisional accession series number GSE31681.

### Microarray data analysis

To compare the gene expression profile of the 24 CC samples according to the oocyte maturation stage, we first filtered the samples based on the “detection call” (i.e., absent/present). Probe sets were used when they were present in at least 7 samples out of 24. A second filter that uses the variation coefficient (40%) between all the samples was also applied. To compare groups of CCs at different stages of oocyte nuclear maturation, a Significance Analysis of Microarrays-Multi-class (SAM-M) [14] was performed. This algorithm provides the score values and a false discovery rate (FDR) confidence percentage based on data permutation. SAM-M allowed the identification of genes whose expression varied significantly among the CC_GV_, CC_MI_ and CC_MII_ categories.

The SAM-M results were used to perform a supervised hierarchical clustering, based on the expression level of the probe sets (multiclass gene set), and the cluster was visualized using the Tree View software [15].

### Reverse-Transcription quantitative Polymerase Chain Reaction (RT-qPCR)

We performed RT-qPCR to validate the expression of the candidate genes using the Superscript First Strand Synthesis System (Invitrogen) according to the manufacturer's recommendation. An independent cohort of CC samples was used for the validation. Strand cDNA was generated starting from 300 ng of total RNA from each sample and used (dilution 1∶10) to assess gene expression by qPCR in 384-wells plates on a Light Cycler 480 (Roche) as described in [16]. Details of the primers used are reported in Table S1. Normalization was performed using the Glyceraldehyde 3-Phosphate Dehydrogenase (*GAPDH*) housekeeping gene.

### Embryo outcome in relation to MII CC gene expression profile

The embryo outcome on day 5 or day 6 of fertilized oocytes has been performed in relation to their gene expression profile of CCs.

### Statistical analysis

The data obtained by RT-qPCR was analyzed with the GraphPad Instat software (http://www.graphpad.com/instat/instat.htm) using the Kruskal-Wallis non-parametric test. The differences among groups were considered significant when the p-value was <0.05.

## Results

### Identification of sets of genes over-expressed in CCs according to each stage of oocyte nuclear maturity

**Figure 1:**
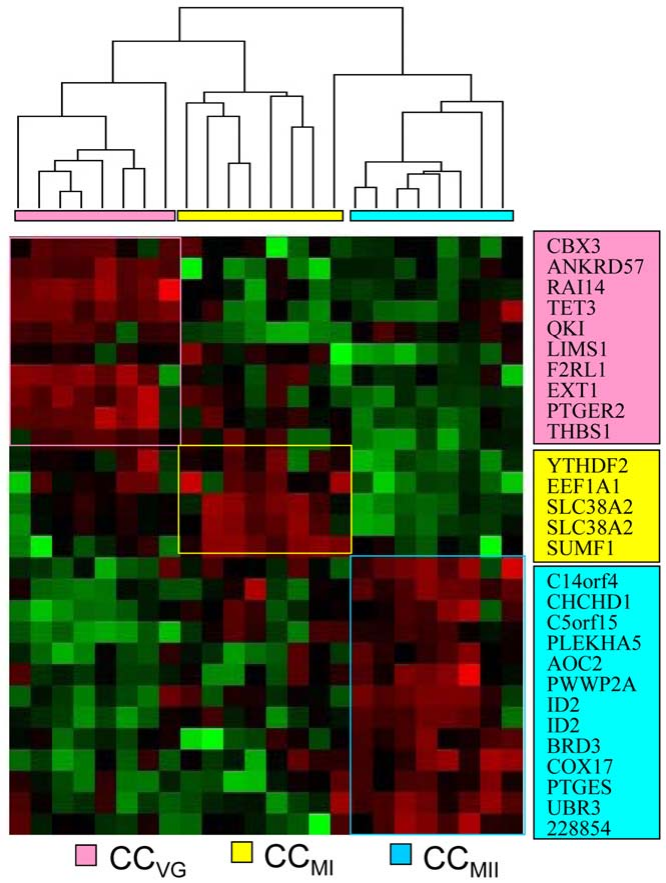
cluster of genes over-expressed in human cumulus cells. This figure shows the supervised hierarchical clustering of genes over-expressed in cumulus cells (CCs) according to the stage of oocyte nuclear maturation. We can see a distinct signature in each CCs category. In red, over-expressed genes; in green, under-expressed genes. CC_GV_, CC_MI_, CC_MII_, CCs issued from oocyte at GV, MI and MII stages respectively.

Using SAM-M, we identified a total of 25 genes (multiclass gene set) with a FDR≤3.30 that significantly distinguished the three groups. These 25 genes were differentially over-expressed according to the stage of nuclear maturity of the associated oocyte. Totally, 10, 4 and 11 genes were specifically over-expressed in the CC_GV_, CC_MI_ and CC_MII_ categories respectively (Table 1). The number of genes that are specific for a given category of CCs indicates that there is a significant variation across the three categories of CCs as demonstrated also by the supervised hierarchical clustering which shows a clear segregation of the CC samples based on this list of 25 genes (Fig. 1). For complete name of these 25 genes, see table 1.

**Table 1:** Candidate transcripts expressed in human cumulus cells.

Probeset	Gene symbol	Gene title	DNA chip p-value	Identified in follicular cells	Species	References
202052_s_at	RAI14	retinoic acid induced 14	0.00195	No	Human	Current study
235542_at	TET3	tet oncogene family member 3	0.00024	No	Human	Current study
201091_s_at	CBX3	chromobox homolog 3 (HP1 gamma homolog, Drosophila)	0.00024	No	Human	Current study
213506_at	F2RL1	coagulation factor II (thrombin) receptor-like 1	0.00024	CCs	Bovine	[18]
201109_s_at	THBS1	thrombospondin 1	0.00024	CCs	Human	[20]
				GCs	Human	[21]
207198_s_at	LIMS1	LIM and senescent cell antigen-like domains 1	0.00073	No	Human	Current study
201995_at	EXT1	exostoses (multiple) 1	0.00073	CCs	Human	[20]
				CCs	Human	[22]
				CCs	Bovine	[18]
219496_at	ANKRD57	ankyrin repeat domain 57	0.00073	No	Human	Current study
206631_at	PTGER2	prostaglandin E receptor 2 (subtype EP2), 53 kDa	0.00024	CCs	Human	[20]
				CCs	Human	[23]
				CCs	Mouse	[24]
				CCs	Mouse	[25]
				GCs	Monkey	[26]
				GCs, CCs	Mouse	[27]
212265_at	QKI	quaking homolog, KH domain RNA binding (mouse)	0.00024	No	Human	Current study
220924_s_at	SLC38A2	solute carrier family 38, member 2	0.00024	No	Human	Current study
213614_x_at	EEF1A1	eukaryotic translation elongation factor 1 alpha 1	0.00024	CCs	Bovine	[18]
226850_at	SUMF1	sulfatase modifying factor 1	0.00073	No	Human	Current study
222430_s_at	YTHDF2	YTH domain family, member 2	0.00024	No	Human	Current study
223474_at	C14orf4	chromosome 14 open reading frame 4	0.00024	GCs	Mouse	[28]
201566_x_at	ID2	inhibitor of DNA binding 2, dominant negative helix-loop-helix protein	0.00024	GCs	Porcine	[29]
				GCs	Hen	[30]
				GCs, TCs	Sheep	[31]
				CCs	Bovine	[18]
203880_at	COX17	COX17 cytochrome c oxidase assembly homolog (S. cerevisiae)	0.02392	No	Human	Current study
226896_at	CHCHD1	coiled-coil-helix-coiled-coil-helix domain containing 1	0.00122	No	Human	Current study
230029_x_at	UBR3	ubiquitin protein ligase E3 component n-recognin 3 (putative)	0.00073	No	Human	Current study
220952_s_at	PLEKHA5	pleckstrin homology domain containing, family A member 5	0.00024	No	Human	Current study
207064_s_at	AOC2	amine oxidase, copper containing 2 (retina-specific)	0.00024	No	Human	Current study
226720_at	PWWP2A	PWWP domain containing 2A	0.00195	No	Human	Current study
210367_s_at	PTGES	prostaglandin E synthase	0.00073	GCs, TCs	Monkey	[32]
				GLCs	Human	[32]
228805_at	C5orf25	chromosome 5 open reading frame 25	0.00024	No	Human	Current study
203825_at	BRD3	bromodomain containing 3	0.00024	CCs	Bovine	[18]

This table shows the genes differentially expressed in human. These data indicates that for each stage of oocyte nuclear maturity, the molecular signature is different in CCs. GCs, granulosa cells; GLCs, granulosa luteal cells; TCs, thecal cells; CCs, cumulus cells.

**Figure 2:**
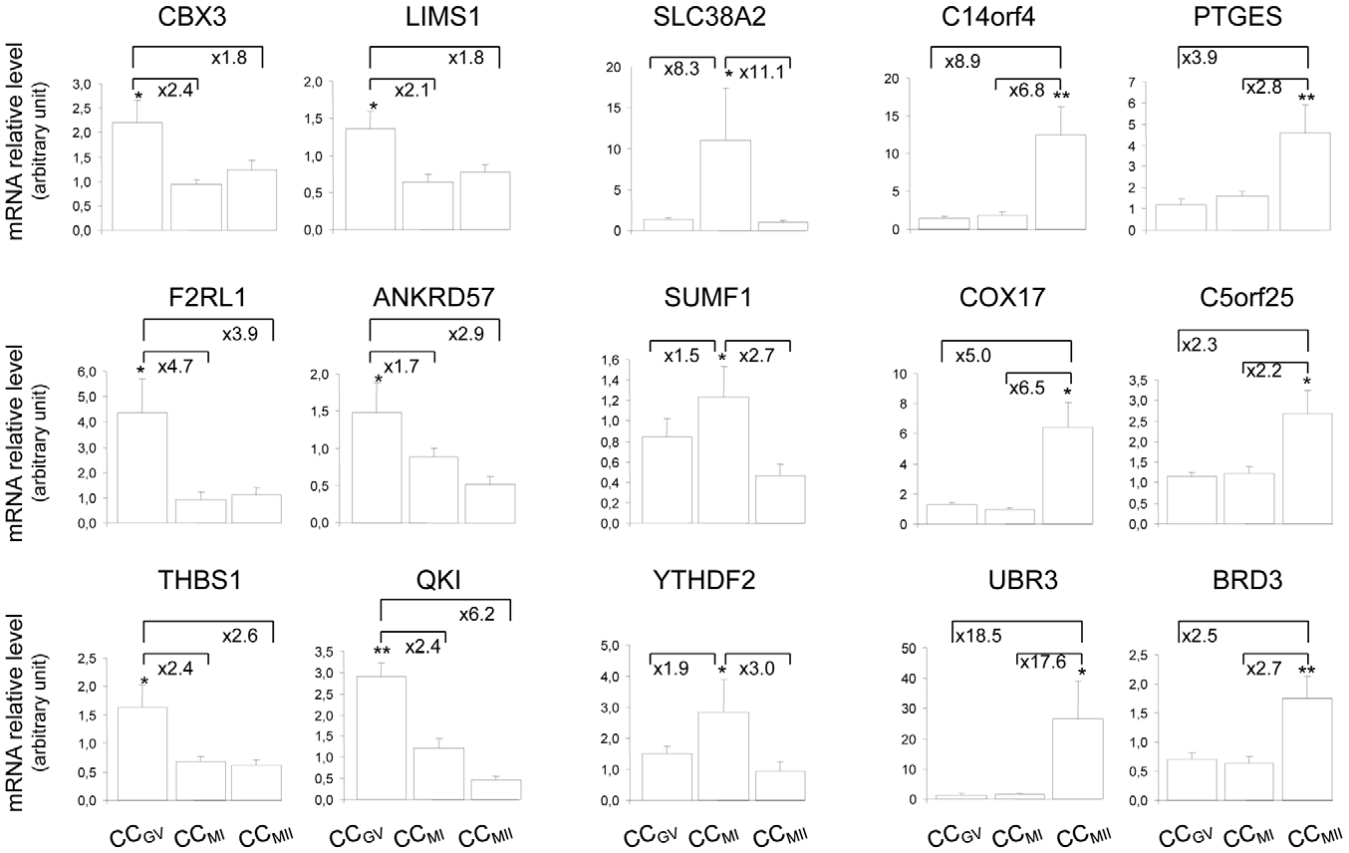
Quantitative RT-PCR confirmation of the microarray data. This figure shows the mRNA relative abundance of genes that were differentially expressed in CCs issued from oocytes at different stages of nuclear maturation. The signal intensity for each gene is shown on the y axis in arbitrary units determined by RT-qPCR analysis. *Indicates a significant difference of gene expression between CCs categories (**p<0.01, *p<0.05). Results were presented as the mean ± SEM.

The 25 genes were then screened by RT-qPCR using an independent cohort of CC samples to strongly validate the microarray results. Fifteen genes were statistically validated as being differentially expressed in the three categories (Fig. 2).

### Metaphase II mature oocytes present distinct expression patterns in their surrounding CCs and embryo outcome

The gene expression profile of 53 CCs isolated from fertilized oocytes has been established. 50% of fertilized oocytes present a CC molecular signature corresponding to CCs at VG or MI stages. The blastulation rate on day 5 or day 6 was more higher in fertilized oocytes surrounded by mature CCs compared with fertilized oocytes surrounded by immature CC gene expression profile (62 *vs.* 17%, P<0.05 respectively).

## Discussion

Cross-talk between CCs and oocyte plays a pivotal role during oocyte maturation. In this study, we identified several genes that are differentially expressed in CCs associated with an oocyte at the GV, MI, or MII stage. These molecular signatures showed that mature oocytes could be surrounded with CCs presenting distinct gene expression profiles.

### Gene expression profile of CCs according to oocyte nuclear maturation stages

There are only few genes differentially expressed in human CCs according to oocyte nuclear maturation stages. This finding is not really surprising for several reasons. First, the current study focuses on a fine biologic question in the same type of cells. Secondly, several studies reported differences in CC gene expression profile according to patients and treatments characteristics [12], [17], probably limiting the observed differences. On the other hand, this is the first study on a large cohort of human CCs comparing differential gene expression profile of individual CCs at each stage of oocyte nuclear maturation under COS and using a transcriptomic global approach. All reported papers in this topic were often restricted to one or two oocyte nuclear maturation stages, and targeted, for the majority, some known genes [18], [19], [20]–[32]. Indeed, CC genes previously described to be related to oocyte nuclear maturation [7], oocyte developmental potential [6], [8]–[11] or embryo development [5] were expressed, but not differentially expressed between our three CC categories.

### Mature oocytes can be surrounded by distinct CC gene expression profiles

Concomitantly with oocyte nuclear maturation, we observed that CCs undergo a molecular maturation process. Our findings demonstrate that mature MII oocyte can be surrounded by either CCs corresponding to CC_VG_, CC_MI_ or CC_MII_ stage respectively. Although the notion of synchronized maturation during folliculogenesis between oocyte and CCs is well documented in mammalian models, it was not yet clearly demonstrated in humans [19]. In the present study, we observed in an independent CC cohort that less than 50% of mature oocytes were surrounded by CCs displaying CC_MII_ signature.

### Oocyte quality associated with mature CCs

We observed a high blastulation rate issues from MII oocyte surrounded by CCs over-expressing CC_MII_. Inversely, mature oocytes over-expressing CC_GV_ or CC_MI_ signature were related to poor blastocyst formation rate. To test the CC status (mature or immature) is thus of a major importance in case of IVF/ICSI failure. In practical value, the quantitative mRNA expression of our CC signatures must rapidly be performed by RT-qPCR (<4 hours) and can help to select the best oocyte quality. Another best way to rapidly (<1 hour) develop and produce assays with high specificity and sensitivity consists in the determination of the quantity of proteins encoded by said genes using a particularly relevant novel approach that combines the use analytical chromatography with a new highly selective mass spectrometry technique called MRM^3^ (MRM, Multiple Reaction Monitoring; Applied Biosystems, SCIEX QTRAP^R^ 5500). This technology is compatible in terms of reproducibility and robustness with a clinical application.

In summary, this study highlights the distinct gene signature of individual CC samples isolated from oocytes at GV, MI and MII stages. Assessing the expression of such signatures is a necessary first step to qualify the CC status as competent or incompetent. CCs screening at the mature oocyte stage is likely to be an accurate tool for detecting competent CCs and may permit the identification of oocyte competence during IVF/ICSI cycles. In addition, these molecular signatures are relevant to elucidate the embryo disorders and IVF failure independently to morphology aspects.

## Supporting Information

Table S1 **Sequences of the primers used for RT-qPCR quantification.**
(DOCX)
