# Supplementary material for: Human Cumulus Cells Molecular Signature in Relation to Oocyte Nuclear Maturity Stage
Source: PLoS One. 2011 Nov 7;6(11):e27179. doi: 10.1371/journal.pone.0027179 (PMC3210145; doi:10.1371/journal.pone.0027179)
Supplement: Table S1 — Sequences of the primers used for RT-qPCR quantification. (DOCX) [file pone.0027179.s001.docx]

Table S1

| **Gene symbol** | **Accession number** | **Primer 5'→3'** | **Fragment size (Bp)** |
| --- | --- | --- | --- |
|  |  | Forward (F) |  |
|  |  | Reverse (R) |  |
| **RAI14** | NM_015577 | F- GCCGCCTCGATGGGGTGTTG | 198 |
|  |  | R- CACTGGCCCCCTTCTTGCCG |  |
| **TET3** | NM_144993.1 | F- GACCTGGCCACCGAAGTCGC | 163 |
|  |  | R- GGGCGTGGGCACAGAAGTCC |  |
| **CBX3** | NM_007276.3 | F- AGTTTCCCTAGCGGGCCATTCC | 149 |
|  |  | R- TGACAGCAGGTCCTAAACTGCC |  |
| **F2RL1** | NM_005242.4 | F- CTTTGGGAGGCTGAGGCAGGC | 196 |
|  |  | R- TCGGCTCACTGCAACCTCTGC |  |
| **THBS1** | NM_003246.2 | F- GCTCACAGCCCTTCGGCCAG | 168 |
|  |  | R- AGCCAGGCTTGCACTCGCAG |  |
| **LIMS1** | NM_004987 | F- GGAGCTGAAAGGGGAGCTATAC | 177 |
|  |  | R- ATGGCGATGTCCAAGAAAGG |  |
| **EXT1** | NM_000127 | F- TCAACACCATCCCTCCTCTC | 165 |
|  |  | R- GTGCTTTTGCCAGTCTTTGC |  |
| **ANKRD57** | NM_023016 | F- GGGGTTACACCGCCCTGCAC | 196 |
|  |  | R- GCGCTTTCCCCGTCACCCTC |  |
| **PTGER2** | NM_000956 | F- CCTGCAGCTGTACGCCACCC | 201 |
|  |  | R- AGGTGGTCCGTCTCCTCCGC |  |
| **QKI** | NM_206855.2 | F- CCTCCAGGGCCCGAAGCTGG | 199 |
|  |  | R- TAGTTGCCGGTGGCGGCTGG |  |
| **SLC38A2** | NM_018976 | F- GCTCGCGCCTTTCCCTTGAC | 197 |
|  |  | R- GAGTCGGCCTCGGTGGTCTC |  |
| **EEF1A1** | NM_001402.5 | F- GATTACAGGGACATCTCAGG | 194 |
|  |  | R- TATCTCTTCTGGCTGTAGGG |  |
| **SUMF1** | NM_001164674.1 | F- TGCTGCTGCTGCTCTCGCTG | 195 |
|  |  | R- GTTGCCGCTCTCCGGGTACG |  |
| **YTHDF2** | NM_001173128.1 | F- ACTCAGGAAGTGCCTCTGGA | 161 |
|  |  | R- GCCTTTTATTTCCCACGACC |  |
| **C14orf4** | NM_024496.2 | F- TGCTGGTGGTAAGAGGCCCG | 196 |
|  |  | R- AGCGAACCTCGTAGGGCGTG |  |
| **ID2** | NM_002166.4 | F- GCTAGCTCAGCAGGCGGCAG | 156 |
|  |  | R- CTCCGGGAGATGCCCAGGCTG |  |
| **COX17** | NM_005694 | F- GACTCAAACCCTGCCCCGCC | 160 |
|  |  | R- GGGCTCTCATGCATTCCTTGTGGG |  |
| **CHCHD1** | NM_203298.2 | F- CGCGGAAGCCTGTGCTGAAG | 187 |
|  |  | R- CGCGGCACAATCGAGGAAGC |  |
| **UBR3** | NM_172070.3 | F- TACGACGAGTTCTGCGCGGC | 152 |
|  |  | R- GTGGTCGCCCTGGTGGAAGC |  |
| **PLEKHA5** | NM_019012 | F- GGCGGCGGATCTGAACCTGG | 150 |
|  |  | R- TGTCCGGTGACCACCGCCTC |  |
| **AOC2** | NM_009590 | F- CACGGCAGGTCTCTGCGTCG | 159 |
|  |  | R- GCACCACCAGTGATGGCGAGG |  |
| **PWWP2A** | NM_052927.2 | F- ACTGACGGGCAGCAATCCGC | 203 |
|  |  | R- GGCGGCGATGCAGGAGAAGG |  |
| **PTGES** | NM_004878.4 | F- CTCCCGGCCTTCCTGCTCTGC | 177 |
|  |  | R- CCCTGAGGCAGCGTTCCACG |  |
| **C5orf25** | NM_198567.4 | F- TGGACAGGACCCCCACCTGC | 200 |
|  |  | F- TGGACAGGACCCCCACCTGC |  |
| **BRD3** | NM_007371 | F- AGGCTTGCGGGTGGACATGC | 153 |
|  |  | R- AACGCGTGAGCAGGGAGCAC |  |
